# Application of the Japanese Verbal Learning Test to Patients With Alzheimer’s Disease in the Early Stage

**DOI:** 10.7759/cureus.62258

**Published:** 2024-06-12

**Authors:** Mie Matsui, Tadasu Matsuoka, Michio Suzuki

**Affiliations:** 1 Laboratory of Clinical Cognitive Neuroscience, Institute of Liberal Arts and Science, Kanazawa University, Kanazawa, JPN; 2 Department of Psychiatry, Matsuoka Hospital, Oyabe, JPN; 3 Department of Neuropsychiatry, University of Toyama, Toyama, JPN

**Keywords:** cdr, delay recall, explicit memory, alzheimer’s disease, verbal learning

## Abstract

Introduction: This study aimed to investigate the mechanism of memory function in the context of explicit memory in early-stage Alzheimer’s disease (AD) using the short-form Japanese Verbal Learning Test (JVLT-9).

Methods: Participants were 20 patients with early-stage AD and a control group of 23 healthy older adults (normal controls: NC), each of whom was administered the JVLT-9, which is a verbal list learning task used to assess explicit memory comprehensively. Between-group differences for each score were investigated using the Mann-Whitney U test. A two-way analysis of variance (ANOVA) was performed for the number of correct recalls by group (AD/NC) × JVLT-9 task. In addition, the AD group was divided into a CDR 0.5 group and a CDR 1.0 group, and it was performed as a group (CDR 0.5/1.0) × JVLT-9 task two-way ANOVA.

Results: The results demonstrated that the AD group had lower immediate recall, learning rate, semantic clustering, and recognition discrimination and significantly higher intrusion errors compared to the NC group. Further, JVLT-9 recall and recognition rates were found to be lower with higher CDR (an index of dementia severity).

Conclusion: These results are largely consistent with the features of explicit memory in AD reported in the English version, confirming the clinical utility of the JVLT-9 as a test of explicit memory function.

## Introduction

Core symptoms of cognitive impairment in Alzheimer’s disease (AD) include memory impairment, aphasia, apraxia, agnosia, and executive dysfunction. Among these, memory impairment is the first to appear and the most marked. Specifically, episodic memory is memories of one’s own experiences, and the ability to answer questions of “when” and “where"-is known to decline early on in the progression of AD. For example, when presented with delayed-recall tasks in tests of cognitive function, individuals with AD may be unable to remember even having been presented with the stimulus, as though the episode they experienced had been deleted from memory [1]. Episodic memory is classified as a type of explicit memory (declarative memory) alongside semantic memory [2]. Recall and recognition tasks are widely used as tools to measure this so-called explicit memory function in both healthy individuals and people with AD. One of the most common among these, primarily used in North America, is Delis, Kramer, Kaplan, and Ober’s [3] California Verbal Learning Test (CVLT), which measures verbal learning and memory. With the CVLT, the subject is presented with a list of words and performs recall and recognition tasks, much like other tests. However, the CVLT was designed to present qualitative indicators of learning and memory, unlike other cognitive function tests that only score overall ability [4]. The term qualitative indicator refers to elements, such as learning strategies or processes and error categories, that are believed to reflect trends in the examinee’s responses [5]. A study by Delis et al. [6] suggested that impaired immediate recall, a low learning rate, inconsistent recall, low semantic clustering, a recency effect, a high intrusion rate, no improvement in delayed recognition, and low recognition discrimination are CVLT findings characteristic of AD. Greenaway et al. [7] compared verbal memory between a mild cognitive impairment (MCI) group, an AD group, and a normal control (NC) group using the CVLT. They found that the MCI group CVLT profile was extremely similar to that of the AD group, which supports the opinion that, in many cases, MCI is a pre-clinical stage of AD. Delis et al. [8] went on to create a revised edition of the CVLT known as the California Verbal Learning Test-Second Edition (CVLT-II). The CVLT-II short form was added with this revision for the first time. It uses a list of nine words and was created for individuals with a brain injury or older adults for whom it may be difficult to administer the standard version, which uses a list of 16 words. An analogous Japanese-language version known as the Japanese Verbal Learning Test (JVLT [9]) is available. Comprehensively evaluating explicit memory, including episodic memory, in early-stage AD using the short form of the JVLT (Japanese Verbal Learning Test 9: JVLT-9) is likely to be of clinical significance. The Clinical Dementia Rating (CDR) is an informant-based global assessment scale with established reliability and validity that has been widely utilized as a dementia severity-ranking scale due to Alzheimer's disease in many studies [10]. We examine features of explicit memory impairment in early-stage AD, which is a type of dementia, and the relationship between CDR [11] and explicit memory function by measuring indicators derived from the JVLT-9, such as recall, semantic clustering, and errors.

This study aims to investigate the features of explicit memory impairment in early-stage AD by conducting a comprehensive assessment of explicit memory, including episodic memory, using the short-form Japanese Verbal Learning Test (JVLT-9). It also investigates the relationship between dementia severity and explicit memory through a comparison of CDR and JVLT-9 results. This study seeks to verify the following hypotheses: First, concerning explicit memory measured by the JVLT-9, similar to the results of Delis et al. [6], we expect to see impaired immediate recall, a low learning rate, inconsistent recall, low semantic clustering, a recency effect, low retention with a delay interval, a high intrusion rate, and low recognition discrimination in individuals with AD. Second, we expect JVLT-9 results to be lower in more severe cases (with a higher CDR score).

## Materials and methods

The study design was a cross-sectional observational study comparing patients and healthy controls. The participants were a group of diagnosed patients and a group of healthy controls matched for age and estimated premorbid IQ.

Participants

The early-stage Alzheimer’s disease group (AD group) consisted of 20 individuals (7 men, 13 women) who had been examined at the Toyama University Hospital and diagnosed by either a neuropsychiatrist or neurologist using the DSM-IV “Dementia of the Alzheimer’s Type” and ICD-10 “Alzheimer’s disease” diagnostic criteria, as well as magnetic resonance imaging (MRI) and single photon emission computed tomography (SPECT). CDR in the AD group was 0.5 (questionable dementia) in 12 cases and 1.0 (mild dementia) in eight cases (average 0.7 ± 0.3 [SD]). Two of the CDR 0.5 patients did not completely satisfy the DSM-IV or ICD-10 diagnostic criteria but were diagnosed with amnestic MCI and included in the AD group due to memory impairment and imaging findings, which are characteristic of AD (e.g., hippocampal atrophy on MRI, decreased parietal lobe blood flow on SPECT). In the AD group, age ranged from 46 to 83 years (average age 70.6 ± 9.2 [SD] years; average years of education 11.5 ± 2.9 [SD] years; average estimated IQ measured by the JART-25 96.8 ± 13.2 [SD]); and the Mini-Mental State Examination (MMSE) score ranged from 15 to 28 points (average score 23.2 ± 3.2 [SD] points). According to the DSM-IV diagnostic criteria, categories (1) other central nervous system conditions that cause progressive deficits in memory and cognition (e.g., cerebrovascular disease, Parkinson’s disease, Huntington’s disease, subdural hematoma, normal-pressure hydrocephalus, brain tumor), (2) systemic conditions that are known to cause dementia (e.g., hypothyroidism, vitamin B12 or folic acid deficiency, niacin deficiency, hypercalcemia, ceurosyphilis, HIV infection), and (3) substance-induced conditions were excluded.

For the normal control group (NC group), volunteers were recruited from Toyama City. Twenty-four applications were received, but one applicant was excluded due to an MMSE score far below the normal range. Ultimately, participation was requested from 23 healthy older adults (13 men, 10 women) aged between 60 and 87 years (average age 74.0 ± 6.0 [SD] years; average years of education 9.8 ± 2.2 [SD] years; average estimated IQ 97.8 ± 13.1 [SD]). Healthy controls were determined to have no difficulties with daily activities, to be adequately functioning in the local community, and to have no history of psychiatric or neurological disorder through a medical interview and questionnaire. CDR in the NC group was 0 for all members. The MMSE score ranged from 24 to 30 points (the MMSE average score was 27.4 ± 1.5 [SD]). Average age and estimated IQ did not differ significantly between the AD and NC groups.

A researcher gave all participants an explanation of the study, either orally or in writing, and obtained their consent. This study was conducted with approval from the Research Ethics Board of Toyama University.

Japanese verbal learning test 9 words version (JVLT-9)

The JVLT-9 is the short form of the JVLT. The scoring method and other elements of the JVLT-9 reference the California Verbal Learning Test second edition (CVLT-II [8]) short form (a shortened version for older adults or those with evident memory impairment). The JVLT-9 includes the following sub-test measures and was predicted to be valid based on previous studies with the same configuration [9,12]. For recall tasks: (1) immediate free recall of a 9-word list (3 words × 3 categories; four trials), (2) short-delay recall (one trial), (3) long-delay recall (one trial), and (4) cued recall (one trial). For recognition tasks: (5) long-delay “yes-no” recognition (one trial), and (6) long-delay forced choice recognition (one trial; optional). Recall tasks are used to calculate the following indicators: (1) number of correct recalls in all trials; (2) semantic clustering score: number of words from the same category answered consecutively; and chance-adjusted semantic clustering: relationship of the actual clustering score to the semantic clustering score expected from the number of correct recalls; (3) serial clustering score: number of words answered in the order that they were presented; and chance-adjusted serial clustering: relationship of the actual clustering score to the serial clustering score expected from the number of correct recalls; (4) recall primacy/recency effects: percentage of correctly recalled words due to differences in presentation order; (5) response errors (number of repetitions: number of times the same response was repeated; number of intrusions: number of incorrect responses that are synonyms of list words, number of incorrect responses that are in the same category as list words, number of incorrect responses that are unrelated to list words); (6) immediate free-recall learning curve: average number of new words acquired in each trial; and (7) consistency of correct recall on immediate free recall: rate at which the same word is answered consistently. Meanwhile, recognition tasks are used to measure the following indicators: (1) the number of recognitions (hits); (2) the number of false recognitions (false alarms: FAs); (3) discriminability (d’): indicator of hit and FA discrimination; and (4) yes-no response bias (criterion: C): indicator of bias toward responding with either “yes” or “no.”

The JVLT-9 uses five categories for which healthy older adults could generate a large number of words (plants, tools, foods, objects, and natural phenomena) based on Sakuma et al. [13]. A further six categories (trees, vegetables, clothing, spices, vehicles, and weather) were selected from among the sub-categories therein. Then, three words showing high character and phonetic familiarity [14] in each of these six categories were selected from among words generated by college students in Matsui and Nakatsubo [15]. These six categories were further divided into two sets consisting of three categories each, with one used as an alternative version. Last, 18 distractor words to be used in long-delay yes-no recognition (prototypical: in the same category as the target word, 3 categories × 3 words; unrelated: in a category unrelated to the target word, nine words) were also selected from Matsui and Nakatsubo [15] (Table 1).﻿

**Table 1:** Words selected for JVLT-9 The word stimuli included in the two versions are shown. Each word is shown as it is pronounced in Japanese, and the English translation is shown in parentheses. The values provided are word familiarity (Min1.0-Max7.0). The stimulus words of JVLT9 are words with high familiarity (5.4 or higher) in the Japanese database [12].

JVLT-9 (A version)					
list	word familiarity	recognition	word familiarity	recognition	word familiarity
		prototypical		unrelated	
take (bamboo)	6.25	sugi (cedar)	6.15	raion (lion)	6.46
piman (green paper)	6.59	buna (beech)	5.18	odeko (forehead)	6.28
ame (rain)	6.56	hinoki (Japanese cypress)	5.47	tetsu (iron)	6.4
kyabetsu (cabbage)	6.56	tomato (tomato)	6.4	kutibeni (lipstick)	6.15
taifuu (typhoon)	6.5	tamanegi (onion)	6.43	teburu (table)	6.46
sakura (cherry blossoms)	6.46	kyuuri (cucumber)	6.4	bukkyo (Buddhism)	6.06
yuki (snow)	6.46	kaminari (thunder)	6.03	zassi (magazine)	6.25
ityou (ginkgo)	6.18	jisin (earthquake)	6.25	mado (window)	6.28
jyagaimo (potato)	6.53	tsunami (tsunami)	5.71	surippa (slipper)	6.34
Mean/SD	6.45/0.14	Mean/SD	6.07/0.40	Mean/SD	6.30/0.13
JVLT-9 (B version)					
list	word familiarity	recognition	word familiarity	recognition	word familiarity
		prototypical		unrelated	
sukato (skirt)	6.53	kutsushita (socks)	6.15	kome (rice)	6.62
satou (sugar)	6.59	boushi (hat)	6.09	mannga (comics)	6.56
densya (train)	6.5	jyaketto (jacket)	6.37	tonnbo (dragonfly)	6.4
sio (salt)	6.46	sake (liquor)	6.37	atama (head)	6.37
takusi (taxi)	6.46	su (vinegora)	6.03	nihonnjinn (Japanese)	6.53
zubon (pants)	6.43	sosu (sauce)	6.34	doa (door)	6.5
basu (bus)	6.53	hikouki (plain)	6.25	chikyuu (earth)	6.62
syatu (shirt)	6.4	fune (ship)	6.28	koutya (black tea)	6.4
miso (miso)	6.28	kuruma (car)	6.5	yubiwa (ring)	6.25
Mean/SD	6.46/0.08	Mean/SD	6.26/0.14	Mean/SD	6.47/0.12

Procedure

For the AD group, the MMSE, JVLT-9, and JART-25 [16] were administered in an outpatient examination room at the hospital. For the NC group, the MMSE, JVLT-9, and JART-25 were administered in a psychology laboratory at the University of Toyama Collaborative Research Building for Medical and Pharmaceutical Science. Conducting all the tests took approximately 40 minutes.

JART-25 is the short version of JART assessing premorbid intellectual ability and has been validated [16]. JART consists of both regular and irregular Japanese words (kanji), which are ideographic scripts. Participants were required to read the words, and the premorbid IQ was evaluated based on the number of errors in reading performance.

The JVLT procedure is as follows: For immediate free recall, an experimenter read a list of nine words aloud and then immediately asked the participant to state all the words they remembered. This was considered one trial and was repeated four times. For short-delay recall, which was performed after completing the four immediate free-recall trials, the participant performed a delay task of counting backward from 100 (e.g., 100, 99, 98...) for 30 seconds and then stated all the words they remembered from the list. For long-delay recall, which was performed after completing short-delay recall, the participant once again stated all the words they remembered from the list after an additional delay of about 20 minutes. For cued recall, which was performed after completing long-delay recall, an experimenter read out the three categories included in the list one at a time, and the participant stated the words that they remembered in each respective category. For long-delay yes-no recognition, an experimenter read out a total of 27 words (nine list words, 18 distractors) in random order, and the participant stated whether or not this was a word from the list for each.

Statistics

Between-group differences for each indicator were investigated using the Mann-Whitney U test. We also performed a two-way analysis of variance (ANOVA) for the number of correct recalls by group (AD/NC) × JVLT-9 task (fourth trial of immediate free recall/short-delay free recall/long-delay free recall/long-delay cued recall/long-delay yes-no recognition).

Last, we divided the AD group into a CDR 0.5 group and a CDR 1.0 group and performed a group (CDR 0.5/1.0) × JVLT-9 task (fourth trial of immediate free recall/short-delay free recall/long-delay free recall/long-delay cued recall/long-delay yes-no recognition) two-way ANOVA.

## Results

JVLT-9 overall results

**Figure 1:**
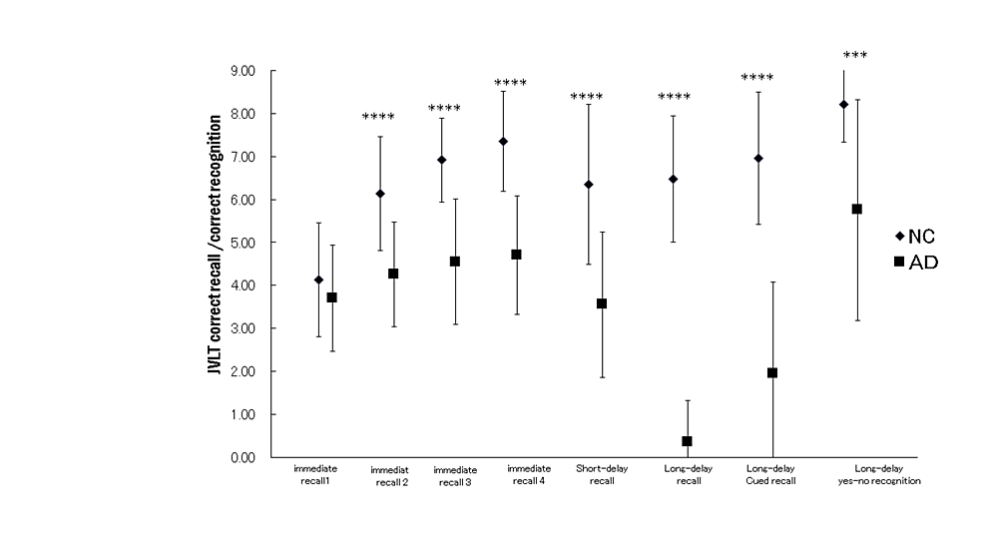
Means of correct recall/correct recognition in JVLT-9 Error bar: SD; NC: normal controls; AD: patients; NC vs. AD: *** p<0.005; **** p<0.001 The learning curve from trial 1 to trial 4 and the subsequent delayed recall and recognition results show that the AD group was lower than the ND group in all scores except immediate recall of trial 1.

The JVLT-9 correct recall and correct recognition averages are shown in Figure 1. Overall, the learning curve from trial 1 to trial 4 and the subsequent delayed recall and recognition results show that the AD group was lower than the ND group in all scores.

Results for each immediate free recall indicator are shown in Table 2. The results of the Mann-Whitney U test revealed that the AD group had significantly fewer correct recalls on the immediate free-recall task than the NC group for the fourth trial and for the total across all trials (fourth trial: p < .001, all trials: p < .001). Semantic clustering was also significantly lower in the AD group than in the NC group for the fourth trial and for the total across all trials (fourth trial: p = .001, all trials: p = .004). Serial clustering was significantly lower in the AD group than in the NC group in the first trial (p = .023). Meanwhile, the chance-adjusted (CA) serial clustering score was significantly higher in the AD group than in the NC group (p = .009). The learning curve score was significantly lower in the AD group than in the NC group (p < .001).

**Table 2:** Results of the immediate free recall U-test, AD vs. NC CA: actual clustering－semantic clustering expected from the number of correct recalls The values provided are JVLT-9 variable scores in immediate free recall. The AD group had significantly fewer correct recalls on the immediate free-recall task than the NC group for the fourth trial and for the total across all trials. Semantic clustering was significantly lower in the AD group than in the NC group for the fourth trial and for the total across all trials. The chance-adjusted (CA) serial clustering score was significantly higher in the AD group than in the NC group.

variable			AD(n=20)		NC(n=23)		p-value
			mean	SD	mean	SD	
correct recall		trial 1	3.7	1.23	4.13	1.33	0.251
		trial 4	4.7	1.38	7.35	1.17	<0.001
		total	17.2	4.58	24.5	3.4	<0.001
semantic clustering		trial 1	0.8	1.08	1.17	0.82	0.088
		trial 4	0.7	0.84	2.22	1.61	0.001
		total	3.3	2.22	6.52	4.12	0.004
semantic clustering CA		1~4	0	0.46	0.35	0.74	0.213
serial clustering		trial 1	0.95	0.67	0.48	0.58	0.023
		trial 4	1.05	0.67	1.17	0.76	0.648
		total	2.6	1.85	4.3	2.87	0.99
serial clustering CA		trial 1~4	0.2	0.4	-0.2	0.91	0.009
error	repetitions	1~4	1.35	1.8	0.74	0.79	0.379
	intrusions	1~4	2.2	3.89	0.61	1.13	0.201
learning slope		1~4	0.33	0.34	1.04	7.3	<0.001

Further, for the number of correct recalls by list position, the AD group had significantly fewer correct recalls compared to the NC group for the middle third and last third (middle third: p = .001, last third: p = .004). The one-way ANOVA conducted for the number of correct recalls by list position in the AD group revealed a main effect for list position, F(2,57) = 17.40, p < .001, and post-hoc testing revealed that correct recalls were significantly lower for the middle third than the first third and last third (p < .001). A main effect for the position was also observed in the NC group, F(2,66) = 5.42, p = .007, and post-hoc testing revealed that correct recalls were significantly higher for the first third than the middle third (p = .009), but significant differences were not observed for other positions (Table 3). 

**Table 3:** Correct recall in presentation order and consistent correct recall U-test, AD vs. NC one-way ANOVA for the number of correct recalls by list position: a main effect for list position, F(2,57) = 17.40, p <0.001 AD: post-hoc test, a: primacy vs. middle, b: middle vs. recency, **: p<0.001 NC: post-hoc test, a: primacy vs. middle, *: p<0.01 The value provided is a number or percentage of correct recall in presentation order. The value of the last line is a number or percentage of consistent correct recall.

Variable			AD (n=20)				NC (n=23)				p-value
			Raw score		Percentage		Raw score		Percentage		
			mean	SD	mean	SD	mean	SD	mean	SD	
Correct recall in presentation order	primacy1/3	1~4	7.05	2.22	41% a **	11%	9.26	2.19	38% a *	8%	0.401
	middle1/3	1~4	3.25	2.43	18% b**	12%	7.3	1.94	30%	7%	<0.001
	recency1/3	1~4	6.8	1.99	41%	10%	7.87	1.94	32%	7%	0.004
Consistent correct recall		1~4	10.05	3.28	58%	10%	14.87	3.27	60%	6%	0.635

Results for each short-delay-free recall indicator are shown in Table 4. The results of the Mann-Whitney U test revealed that the AD group had significantly fewer correct recalls and less semantic clustering compared to the NC group (correct recalls: p < .001, semantic clustering: p = .026). Further, for errors, the AD group had significantly more intrusions than the NC group (p = .003).

**Table 4:** Results of short-delay free recall and long-delay free recall U-test, AD vs. NC CA: actual clustering-semantic clustering expected from the number of correct recalls The values provided are JVLT-9 variable scores in short-delay-free recall and long-delay-free recall. The AD group had significantly fewer correct recalls and less semantic clustering compared to the NC group in both short-delay and long-delay-free recalls.

Variable			AD(n=20)		NC(n=23)		p-value
			mean	SD	mean	SD	
short-delay free recall	correct recall		3.55	1.69	6.35	1.86	<0.001
	semantic clustering		1.05	0.97	2.04	1.52	0.026
	semantic clustering (CA)		0.54	0.72	0.95	1.35	0.341
	serial clustering		0.45	0.59	0.91	1.1	0.206
	serial clustering (CA)		-0.1	0.54	-0.3	1.02	0.311
	error	repetitions	0.1	0.3	0.3	0.46	0.104
		intrusions	1.05	1.53	0.13	0.34	0.003
long-delay free recall	correct recall		0.35	0.96	6.48	1.47	<0.001
	semantic clustering		0.15	0.48	2.13	1.51	<0.001
	semantic clustering (CA)		0.28	0.29	1.01	1.37	0.014
	serial clustering		0.05	0.22	0.87	0.95	0.001
	serial clustering (CA)		0.19	0.11	-0.3	0.91	0.024
	error	repetitions	0	0	0.39	0.77	0.015
		intrusions	0.3	1.1	0.48	0.58	0.024

Results for each long-delay-free recall indicator are also shown in Table 4. The results of the Mann-Whitney U test revealed that the AD group had significantly fewer correct recalls and less semantic clustering, semantic clustering (CA), and serial clustering compared to the NC group (correct recalls: p < .001, semantic clustering: p < .001, semantic clustering [CA]: p = .014, serial clustering: p = .001). Meanwhile, the serial clustering (CA) score was significantly higher in the AD group compared to the NC group (p = .024). Moreover, for errors, the AD group had significantly fewer repetitions and intrusions than the NC group (repetitions: p = .015, intrusions: p = .024).

Short-/long-delay free-recall retention rates are shown in Table 5. Retention rate refers to the number of correct recalls in short-/long-delay free-recall tasks divided by the number of correct recalls in the fourth immediate free-recall trial. This indicator represents the extent to which words learned in the four immediate free-recall trials were retained in delayed-recall tasks. The results of the Mann-Whitney U test revealed that the AD group retention rate was significantly lower for long-delay free recall (p < .001).

**Table 5:** Long-delay retention rate/recognition/errors/correct recall U-test, AD vs. NC The values provided are JVLT-9 variable scores. The AD group retention rate was significantly lower for long-delay-free recall. The AD group had significantly fewer correct recognitions (hits) than the NC group. The AD group had significantly more intrusions of unrelated words than the NC group.

Variable			AD (n=20)		NC (n=23)		p-value
			mean	SD	Mean	SD	
Retention rate	Short-delay free recall		76%	33%	87%	24%	0.206
	Long-delay free recall		6%	16%	89%	19%	<0.001
Cued long-delay free recall	Correct recall		1.95	2.13	6.96	1.55	<0.001
	Error	repetitions	0	0	0.04	0.2	0.351
		intrusions	1.6	1.96	0.57	0.58	0.108
Long-delay yes-no recognition	Recognitions (Hit)		5.75	2.57	8.22	0.88	0.002
	False-alarm		2.65	2.59	0.65	1.05	0.002
	Discriminability: d’		1.57	0.94	2.91	0.48	<0.001
	Yes-no response bias (criterion: C)		0.35	0.59	0.22	0.33	0.533
Errors in all trial recalls	Repetitions		1.45	1.77	1.48	1.47	0.696
	Intrusions	synonym	0	0	0.22	0.72	0.182
		Same category words	2.8	3.64	1.43	1.74	0.34
		unrelated word	2.35	4.75	0.13	0.61	0.005

Results for each long-delay yes-no recognition indicator are also shown in Table 5. Note that a correction was performed for d’ and C calculations. Since the z score required to calculate indicators such as d’ cannot be obtained when the hit rate or FA rate is at 0% or 100%, all data were adjusted by adding 0.5 [17]. The results of the Mann-Whitney U test revealed that the AD group had significantly fewer correct recognitions (hits) than the NC group (p = .002). The AD group also had a significantly higher number of false recognitions (false alarms) compared to the NC group (p = .002). The discrimination indicator (d’) was significantly lower in the AD group than in the NC group (p < .001).

Error results for all recall trials (immediate free recall 1-4, short-/long-delay free recall, and long-delay cued recall) are shown in Table 5. The results of the Mann-Whitney U test revealed that the AD group had significantly more intrusions of unrelated words than the NC group (p = .005).

The number of correct recalls or correct recognitions for each task is shown in Table 6.

**Table 6:** Comparison of correct recall/recognition tasks Note: The values provided are JVLT-9 variable scores. two way ANOVA: group (2) x task (5) main effect for group F(1,41) = 96.25, p <0 .001 main effect for task F(4,38) = 56.32, p < 0.001 group x task interaction F(4,38) = 20.18, p <0 .001 post-hoc tests of AD group immediate recall >short delay recall, p=0.032 immediate recall>long delay recall, p <0.001 immediate recall>cue recall, p <0.001 short delay recall>long delay recall, p <0.001 short delay recall>cue recall, p <0.001 short delay recall<recognition, p <0.001 long delay recall< cue recall, p=0.002 long delay recall<recognition, p <0.001 cue recall<recognition, p <0.001 no significant differences for other tasks post-hoc tests of NC group short delay recall<recognition, p <0.001 long delay recall<recognition, p =0.002 cue recall<recognition, p =0.006 no significant differences for other tasks

Variable		AD (n=20)		NC (n=23)		AC vs. NC
		mean	SD	Mean	SD	p (post-hoc tests of task x group interaction)
Correct recall/ recognition	Immediate free recall trial 4	4.7	1.38	7.35	1.17	<0.001
	Short-delay free recall	3.55	1.69	6.35	1.86	<0.001
	Long-delay free recall	0.35	0.96	6.48	1.47	<0.001
	Cued long-delay free recall	1.95	2.13	6.96	1.55	<0.001
	Long-delay yes-no recognition	5.75	2.57	8.22	0.88	<0.001

We performed group (AD/NC) × task (fourth trial of immediate free recall/short-delay free recall/long-delay free recall/long-delay cued recall/long-delay yes-no recognition) ANOVA. The results revealed significant main effects for group, F(1,41) = 96.25, p < .001, and task, F(4,38) = 56.32, p < .001, and a significant interaction between group and task, F(4,38) = 20.18, p < .001. Multiple comparisons of the task main effect revealed a significantly higher number of correct recognitions for long-delay yes-no recognition compared to the number of correct recalls for the fourth immediate free-recall trial (p < .001), short-delay free-recall (p < .001), long-delay free-recall (p < .001), and long-delay cued-recall tasks (p < .001). Next, significantly more correct recalls were observed for the fourth immediate free-recall trial than for the short-delay free-recall, long-delay free-recall, and long-delay cued-recall tasks (p < .001) and for the short-delay free-recall task than for the long-delay cued-recall task (p < .001). Last, significantly more correct recalls were observed for the long-delay cued-recall task than for the long-delay free-recall task (p<.001).

Post-hoc tests were performed because an interaction was observed between the group and the task. The results revealed simple main effects for group in all tasks: fourth immediate free-recall trial (F = 27.00, p < .001, NC > AD), short-delay free recall (F = 30.15, p < .001, NC > AD), long-delay free recall (F = 144.63, p < .001, NC > AD), long-delay cued recall (F = 96.53, p < .001, NC > AD), and long-delay yes-no recognition (F = 23.45, p < .001, NC > AD).

A simple main effect for the task was also observed in both the AD (F = 68.12, p < .001) and NC (F = 8.370, p < .001) groups. The results of multiple comparisons for tasks in the AD group revealed a significantly higher number of correct recognitions for long-delay yes-no recognition compared to that of correct recalls for short-delay free-recall (p < .001), long-delay free-recall (p < .001), and long-delay cued-recall (p < .001) tasks. Next, significantly more correct recalls were observed for the fourth immediate free-recall trial than for the short-delay free-recall (p =.032), long-delay free-recall (p < .001) and long-delay cued-recall (p < .001) tasks, and for the short-delay free-recall task than for the long-delay cued-recall and long-delay free-recall tasks ((p < .001). Last, significantly more correct recalls were observed for the long-delay cued-recall task than for the long-delay free-recall task (p =.002). The results of multiple comparisons for tasks in the NC group revealed a significantly higher number of correct recognitions for long-delay yes-no recognition compared to that of correct recalls for short-delay free-recall (p < .001), long-delay free recall (p =.002) and long-delay cued-recall (p = .006) tasks, but differences for other tasks were not significant.

CDR and correct recall/recognition for each task

The number of correct recalls or correct recognitions for each task is shown in Table 7. We divided the AD group into a CDR 0.5 group and a CDR 1.0 group and performed a CDR group (0.5/1.0) × task (fourth trial of immediate free recall/short-delay free recall/long-delay free recall/long-delay cued recall/long-delay yes-no recognition) ANOVA. The results revealed significant main effects for group, F(1,18) = 12.89, p < .001, and task, F(4,15) = 49.29, p < .001, and a significant interaction between group and task, F(4,15) = 2.80, p = .003.

**Table 7:** Number of correct recalls/recognitions in each JVLT-9 task for CDR0.5 and 1.0 groups in AD Note: The values provided are JVLT-9 variable scores. two way ANOVA: group (2) x task (5) main effect for group F(1,18) = 12.89, p <0 .001 main effect for task F(4,15) = 49.29, p < 0.001 group x task interaction  F(4,15) = 2.80, p = 0.032 post-hoc tests of the 0.5 group immediate recall>long delay recall, p <0.001 immediate recall>cue recall, p =0.003 short delay recall>long delay recall, p <0.001 short delay recall>cue recall, p =0.046 short delay recall<recognition, p <0.001 long delay recall< cue recall, p=0.024 long delay recall<recognition, <0.001 cue recall<recognition, p <0.001 no significant differences for other tasks post-hoc tests of 1.0 group immediate recall>long delay recall, p <0.001 immediate recall>cue recall, p =0.005 short delay recall>long delay recall, p <0.001 long delay recall<recognition, p =0.003 cue recall<recognition, p =0.003 no significant differences for other tasks

Variable	1.0 group (n=8)		0.5 group (n=12)		0.5 vs. 1.0
	mean	SD	mean	SD	p (post-hoc tests of task x group interaction)
Immediate free recall trial 4	3.75	0.97	5.33	1.25	0.01
Short-delay free recall	2.5	1.32	4.25	1.53	0.022
Long-delay free recall	0	0	0.58	1.19	0.204
Cued long-delay free recall	0.75	1.09	2.75	2.28	0.042
Long-delay yes-no recognition	3.75	1.98	7.08	1.98	0.003

Multiple comparisons of the task main effect revealed a significantly higher number of correct recognitions for long-delay yes-no recognition compared to that of correct recalls for the short-delay free-recall, long-delay free-recall, and long-delay cued-recall tasks (p < .001). Next, significantly more correct recalls were observed for the fourth immediate free-recall trial than for the short-delay free-recall (p = .005), long-delay free-recall (p < .001), and long-delay cued-recall (p < .001) tasks, and for the short-delay free-recall task than for the long-delay free-recall and long-delay cued-recall tasks (p < .001). Last, there were significantly more correct recalls for the long-delay cued-recall task than for the long-delay free-recall task (p < .001).

Post-hoc tests were performed as an interaction was observed between the group and the task. The results revealed simple main effects for group in the fourth immediate free-recall trial (F = 8.28, p = .010, 0.5 group > 1.0 group), short-delay free recall (F = 6.26, p = .022, 0.5 group > 1.0 group), long-delay cued recall (F = 4.82, p = .042, 0.5 group >1.0 group), and long-delay yes-no recognition (F = 12.24, p =.003, 0.5 group > 1.0 group). A simple main effect for the task was also observed in both the CDR 0.5 (F = 35.17, p < .001) and 1.0 (F = 16.92, p < .001) groups. The results of multiple comparisons for the task in the CDR 0.5 group revealed a significantly higher number of correct recognitions for long-delay yes-no recognition compared to that of correct recalls for short-delay free-recall (p < .001), long-delay free-recall (p < .001), and long-delay cued-recall (p < .001) tasks. Next, significantly more correct recalls were observed for the fourth immediate free-recall trial than for the long-delay free-recall and long-delay cued-recall tasks (p < .001), and significantly more correct recalls for the short-delay free-recall task than for the long-delay free-recall (p < .001) and long-delay cued-recall (p = .005) tasks. Last, significantly more correct recalls were observed for the long-delay cued-recall task than for the long-delay free-recall task (p < .001). The results of multiple comparisons for tasks in the CDR 1.0 group revealed a significantly higher number of correct recognitions for long-delay yes-no recognition compared to that of correct recalls for the long-delay free-recall and long-delay cued-recall tasks (p < .001). Moreover, significantly more correct recalls were observed for the fourth immediate free-recall trial than for the long-delay free-recall and long-delay cued-recall tasks (p < .001), and for the short-delay free-recall task than for the long-delay free-recall (p < .001) and long-delay cued-recall (p = .008) tasks. Differences between other tasks were not significant.

## Discussion

This study investigated explicit memory in AD using the JVLT-9. The results demonstrated that the number of correct recalls was significantly lower in the AD group compared to the NC group for the fourth trial, across all trials of the immediate free-recall task, and for all other recall tasks. The same was true for correct recognitions. In AD, correct recall/recognition between each task was as follows: long-delay yes-no recognition > fourth immediate free-recall trial > short-delay free recall > long-delay cued recall > long-delay free recall. Meanwhile, in NC, the order was long-delay yes-no recognition > fourth immediate free-recall trial > short-delay free recall ≒ long-delay free recall ≒ long-delay cued recall. In other words, delay interval length was found to have a large impact on word memory retention in AD. This is consistent with neuropathology findings showing hippocampal atrophy beginning early on in AD [18,19]. The hippocampus is involved in the consolidation of memories, that is, converting short-term memories into long-term ones [20]. This suggests that, in AD, words learned during immediate free recall cannot be converted into long-term memories due to hippocampal degeneration, leading to extremely low correct recall for long-delay free-recall tasks.

Semantic clustering, one key indicator of the JVLT-9, reflects the extent to which words that share semantic features are actively organized together [8]. This learning strategy is said to be an efficient method of encoding long-term memories and creating a “mental filing system” that enables their subsequent retrieval [5]. The semantic clustering score was significantly lower in AD than in NC for the fourth trial and across all trials for immediate free recall, short-delay free recall, and long-delay free recall. In other words, the capacity to use this efficient learning strategy seems to be decreased in AD. However, a large difference may have been observed simply because the number of correct recognitions in the long-delay free-recall task was extremely small. However, in most cases, serial clustering is a relatively inefficient learning strategy, and it correlates with low results for many parameters of the CVLT-II [3]. The serial clustering score was significantly higher in AD than in NC for the first immediate free-recall trial, and the serial clustering CA (relationship of the actual clustering score to the serial clustering score expected from the number of correct recalls) was also significantly higher. This suggests a tendency to use relatively inefficient learning strategies in AD. Serial clustering and serial clustering CA were significantly lower in AD than in NC for the long-delay free-recall task; nevertheless, this may be due to the extremely low number of correct recalls.

Regarding errors, group differences for immediate free recall and long-delay cued recall were not significant; however, the AD group had significantly more intrusions than the NC group for short-delay free recall. Further, looking at errors in all recall trials, the AD group had significantly more unrelated word intrusions than the NC group. Past studies have reported that in cases of marked memory impairment with localized lesions of the left temporal lobe and hippocampus, or AD or Korsakoff’s syndrome, individuals may show even worse intrusion errors as though they are convinced that they have responded correctly from the start [6,21]. Among intrusion errors, the intrusion of unrelated words is said to be the most serious [8]. The present results support these past studies.

The learning curve score throughout the four immediate free-recall tasks was significantly lower in AD than in NC. This suggests that AD participants struggled to learn new words in each trial, which is consistent with the research by Delis et al. [6].

Past studies have shown that the recency effect is more pronounced than the primacy effect in AD due to the ability to easily respond from short-term memory without encoding into long-term memory [22]. In the present study, a recency effect was observed in AD despite a significantly lower correct recall rate compared to NC.

The results of the present study revealed no difference in correct recall consistency between AD and NC throughout the four immediate free-recall trials, a finding inconsistent with the lack of recall consistency in AD reported by Delis et al. [6].

For the long-delay yes-no recognition task, the number of correct recognitions (hits) was significantly lower in AD than in NC, whereas that of false recognitions (false alarms) was significantly higher in AD than in NC. Therefore, the discrimination indicator (d’) was significantly lower in AD than in NC, demonstrating that those with AD were less able to discriminate between list words and distractors in recognition tasks. This is consistent with research by Delis et al. [6]. However, concerning bias toward “yes” responses or “no” responses (C), both groups tended somewhat toward “yes” responses and did not differ from one another, as seen in Delis et al. [6].

To examine differences by AD severity, the AD group was divided into a CDR 0.5 (questionable dementia) group and a CDR 1.0 (mild dementia) group for analysis. The results demonstrated that those with mild dementia had significantly fewer correct recalls than those with questionable dementia. In those with questionable dementia, correct recall/recognition rates were as follows: long-delay yes-no recognition > fourth immediate free-recall trial ≒ short-delay free recall > long-delay cued recall > long-delay free recall. Meanwhile, in those with mild dementia, the order was long-delay yes-no recognition ≒ fourth immediate free-recall trial ≒ short-delay free recall > long-delay free recall ≒ long-delay cued recall. Fleischman et al. [23] found that explicit memory measured by recall and recognition has a negative correlation with AD neuropathology severity. Further, larger senile plaques, a neuropathological feature of AD, in the subiculum and entorhinal cortex have also been reported as having higher CDR [24]. Taking these findings into account, one would expect to see a negative correlation between CDR and explicit memory, which is in line with the results of the present study. Administering the JVLT-9 to both early-stage AD patients and healthy older adults in the present study yielded highly significant findings.

Limitations

This study used a small sample, and future investigations with a wider age range are needed. To further improve the clinical utility of the JVLT-9, it will likely be necessary to perform additional studies with a larger sample size. Accordingly, future research verifying the correspondence between the impairments demonstrated in the memory function tests performed here and brain imaging will contribute to clarifying the neurological mechanisms of memory impairment and improving clinical utility. In clinical practice, a simple memory test to evaluate the presence or absence of memory impairment may not be sufficient. JVLT-9 is one of the clinical tools that incorporates principles from cognitive science to quantitatively measure the multifaceted elements of learning and memory concerning CVLT and was created based on many clinical findings and psychological research on memory. In other words, JVLT-9 has the advantage of being able to score qualitative aspects of memory, such as error types, learning strategies, and mechanisms of memory decline. Previous studies have reported, for example, the characteristics of differences between AD and subcortical dementia [12], and the clinical usefulness of JVLT-9 is considered to be great.

## Conclusions

The JVLT-9 profile of AD is characterized by impaired immediate recall, a low learning rate, low semantic clustering, a high intrusion rate, and low recognition discrimination. This profile is similar to the findings of CVLT. However, neither recall consistency nor recognition response bias differed between the two groups. Thus, our hypothesis that JVLT-9 results for AD would be identical to those of CVLT results was not fully supported. However, regarding the relationship between CDR and JVLT-9, our hypothesis that JVLT-9 results would decline as the CDR score increased was supported by the results, which suggests that the JVLT-9 is highly likely to be useful in clinical settings.
